# The Influence of Maternal Factors on Neonatal Intensive Care Unit Admission and In-Hospital Mortality in Premature Newborns from Western Romania: A Population-Based Study

**DOI:** 10.3390/medicina58060709

**Published:** 2022-05-26

**Authors:** Stelian-Gabriel Ilyes, Veronica Daniela Chiriac, Adrian Gluhovschi, Valcovici Mihaela, George Dahma, Adelina Geanina Mocanu, Radu Neamtu, Carmen Silaghi, Daniela Radu, Elena Bernad, Marius Craina

**Affiliations:** 1 Department of Obstetrics and Gynecology, “Victor Babes” University of Medicine and Pharmacy Timisoara, 300041 Timisoara, Romania; dr.ilyesstelian@yahoo.ro (S.-G.I.); adigluhovschi@yahoo.com (A.G.); george_dahma@yahoo.com (G.D.); adelinaerimescu@yahoo.com (A.G.M.); radu.neamtu@umft.ro (R.N.); silaghi.carmen@gmail.com (C.S.); ebernad@yahoo.com (E.B.); mariuscraina@hotmail.com (M.C.); 2 Department of Cardiology, “Victor Babes” University of Medicine and Pharmacy Timisoara, 300041 Timisoara, Romania; mihaeladanielacardio@gmail.com; 3 Department of General Surgery, “Victor Babes” University of Medicine and Pharmacy Timisoara, 300041 Timisoara, Romania; daniela_radu@hotmail.com

**Keywords:** prematurity, risk factors, premature birth, neonatal intensive care unit, in-hospital mortality

## Abstract

*Background and Objectives*: Neonatal mortality is a global public health issue, disproportionately affecting low- and middle-income nations. Although Romania is a high-income nation, according to the European Union’s most recent demographic data, it had the second-highest infant death rate in 2019. Although significant progress has been made in the last three decades in lowering newborn mortality, more initiatives to accelerate progress are required to meet the 2030 Sustainable Development Goals (SDG) objective. Therefore, we aimed to develop an observational study to determine the influence of maternal factors on in-hospital neonatal intensive care unit admission and mortality in premature infants born in western Romania. While newborn mortality has decreased globally, the pace of decline is far less than what is desired. *Materials and Methods*: A retrospective study comprising 328 premature patients and 422 full-term newborns, was developed at a tertiary obstetrics and gynecology clinic in western Romania, comprising the period of the last 24 months before the COVID-19 pandemic and the first 24 months of the pandemic. *Results*: The following variables were identified as statistically significant risk factors for neonatal intensive care unit admission: age > 35 years, OR = 1.59; twin births, OR = 1.14; low gestational age, OR = 1.66; preeclampsia, OR = 2.33; and peripartum infection, OR = 2.25. The same risk factors, with the exception of twin births, were significantly associated with in-hospital neonatal mortality. Except for a longer duration of maternal hospitalization and neonatal therapy with surfactant, steroids, and antibiotics, the COVID-19 pandemic did not cause significant differences in the evolution and outcomes of preterm newborns. *Conclusions*: The major maternal risk factors for NICU admission were advanced age, twin pregnancy, low gestational age, preeclampsia, and peripartum infection. Additionally, these characteristics contributed to a high likelihood of death, despite adequate access to medical care and advanced life support for the neonates. Understanding the causes of morbidity and death in neonates admitted to the neonatal intensive care unit enables better prioritization and planning of health services, resource reallocation, and care quality improvement.

## 1. Introduction

Neonatal mortality is a public health issue that affects the world’s poorest and middle-income countries the most [1]. Even though significant progress has been made in reducing infant mortality over the last three decades, further efforts are needed to improve survival rates and severe complications [2]. Otherwise, if neonatal mortality is not significantly reduced between 2020 and 2030, the statistics forecast that over 30 million babies will die during this period [3]. The risk of early newborn death is very high across several regions with negative particularities, according to data collected from 186 countries [4]. Roughly half of all newborn deaths happened within 24 h of birth, and about a third occurred within the first 6 h [5].

Even while neonatal mortality is decreasing globally, the rate of decline is far slower than that of post-neonatal death under five years old. The majority of newborn deaths in low- and middle-income nations occur without a known cause of death [6]. Because various factors could be linked to the actual underlying cause of neonatal mortality, it is difficult to confirm the reason; nonetheless, research has classified causes into those related to maternal or fetal disorders [7]. In developing countries, neonatal mortality is frequently caused by illnesses such as tetanus or community-acquired infections that manifest as an emergency soon after birth or later [8,9], while in developed countries such as Romania, the main causes of neonatal deaths are immaturity, congenital defects, and birth injuries [10,11]. Statistics on the causes of newborn deaths and the timing of neonatal deaths are generally scant and less trustworthy than data on all-cause mortality, resulting in unclear estimates, which makes developing evidence-based strategies to reduce neonatal deaths difficult [12]. Improved data on where and when neonatal deaths occur and the factors that cause delays are essential for developing context-specific community and strategy plans [13].

Preterm birth is often regarded as the leading risk factor for perinatal morbidity and death, as 15 million newborns are prematurely born every year [14]. Although there has been a slight decline in preterm births worldwide from the first to the second decade of this millennium, and preterm survival rates have increased in developed countries, preterm neonates continue to die in many underdeveloped countries due to a lack of adequate newborn care [15,16]. Moreover, during the COVID-19 pandemic, pregnant women discontinued their routine prenatal care and more were brought to the obstetric emergency department with problems needing urgent attention, leading to an increase in the number of neonates diagnosed with small for gestational age and hypoxic-ischemic encephalopathy [17]. Also, in a large cohort research study, COVID-19 in pregnancy was related to considerable and persistent increases in severe maternal morbidity and death, as well as negative newborn outcomes, when comparing pregnant women with and without a COVID-19 diagnosis [18]. Therefore, it is critical to identify the risk factors for preterm delivery, as preterm labor and its associated complications have long been a source of concern in the medical community. 

Preterm deliveries have been observed to cause a disproportionate number of newborn fatalities [19]. As a result, prematurity is seen as a significant impediment to achieving the Millennium Development Goals [20]. Even if the preterm newborn survives through the higher risk of infections and other complications of prematurity, it may still have impacts on neurodevelopmental functioning, such as an increased risk of cerebral palsy, delayed learning and/or vision impairments, and chronic illnesses in adulthood [21,22]. Identifying risk factors is the first and most critical step in developing intervention methods aimed at reducing the frequency of preterm births and their associated complications. Some known risk factors are maternal age over 35 years, a family history of preterm births, and membrane rupture [23,24]. Understanding these characteristics and their interplay may result in significant advances in preterm birth diagnosis, prevention, and therapy. Thus, in a socioeconomically developed city in western Romania, this study looked at the contributing factors for neonatal intensive care unit admission and in-hospital mortality among newborns with a gestational age of up to 37 weeks. The secondary endpoint was to compare these factors over a period of the 24 months prior to the COVID-19 pandemic with the 24-month period of the beginning of the COVID-19 pandemic.

## 2. Materials and Methods

An observational study with patients enrolled between 2018 and 2021 was conducted at the University Clinic of Obstetrics and Gynecology “Bega” associated with the “Victor Babes” University of Medicine and Pharmacy in Timisoara, Romania. The research population and relevant features were identified using a population-based administrative database of patients who appeared in the outpatient setting of the same clinic throughout the study period. Our centralized database included patient medical records protected by privacy laws and obtained with the patient’s agreement, including their demographic information, medical history, and in-hospital procedures. The baseline characteristics and procedures for all patients were recorded in the hospital database, as well as in paper patient records inspected by certified clinicians participating in the current study. We performed a computerized database search to ascertain the precise diagnosis as defined by the International Classification of Diseases (ICD-10) and the procedures defined by the Current Procedural Terminology (CPT).

**Figure 1:**
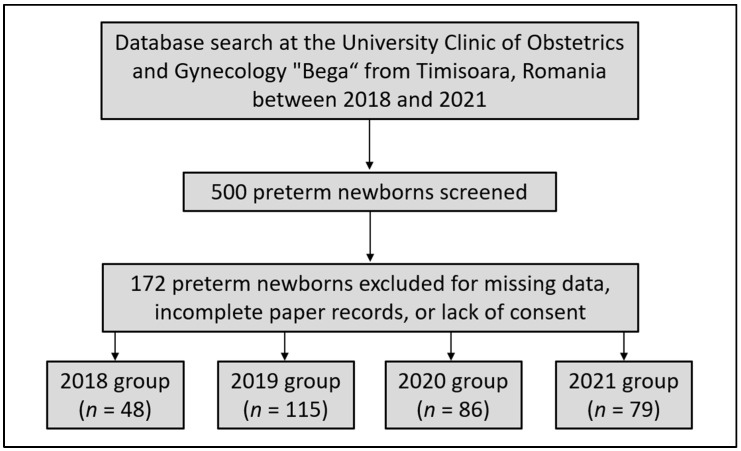
Flowchart displaying preterm newborns included in the current study.

Neonates were included in the current study based on the World Health Organization (WHO) definition of prematurity [25]. Preterm birth is defined as any birth that happens before 37 completed weeks of gestation or less than 259 days after the first day of the woman’s last menstrual period. Neonatal mortality was defined as death occurring in a newborn within the first 28 days of life [26]. Based on the same criteria, severe prematurity was considered for newborns with a gestational age under 28 weeks. Other inclusion criteria comprised a date of birth between 2018 and 2021, and maternal consent for using private medical records of the mother–child dyad. Patients were excluded from the study if medical records were incomplete or missing data of interest, or when the consent was not signed in the existing papers (Figure 1). Using a convenience sampling method, it was determined that a total of 385 cases were adequate for representing each of the preterm newborn group and the full-term newborns.

The Timis County Emergency Clinical Hospital “Pius Brinzeu” Local Commission of Ethics for Scientific Research operates in accordance with Article 167 of Law No. 95/2006, Art. 28, Chapter VIII of Order 904/2006; with EU GCP Directives 2005/28/EC, the International Conference on Harmonisation of Technical Requirements for Registration of Pharmaceuticals for Human Use (ICH); and with the Declaration of Helsinki—Recommendations Guiding Medical Practice. Approval number 23 was assigned to the present research on 20 January 2022.

The variables considered for statistical analysis comprised maternal background data (age, weight at birth, frequency of twin births, gestational age, days of hospitalization, and percentage of cases with high obstetrical risk). A high obstetrical risk pregnancy was considered as any condition associated with a pregnancy where there is an actual or potential risk to the mother or fetus [27]. Data were also collected on maternal comorbid conditions (preeclampsia, thrombophilia, anemia, peripartum infection, and other maternal infections), neonatal characteristics (gender, APGAR score, birth weight, in vitro fertilization, type of delivery, infection after membrane rupture, congenital abnormalities, severe prematurity, NICU admission, resuscitation, days of hospitalization, days of NICU stay, and mortality), and neonatal therapy (surfactant, steroids, and antibiotics). A peripartum infection was considered as “a bacterial infection of the genital tract or surrounding tissues occurring at any time between the onset of rupture of membranes or labour and the 42nd day postpartum”, according to the WHO [28].

IBM SPSS software version 26.0 was used for statistical analysis (SPSS. Inc., Chicago, IL, USA). Absolute and percentage values were used to represent categorical variables. For parametric and non-parametric variables, respectively, the Student’s *t*-test and the Mann–Whitney U-test were employed. A Shapiro–Wilk test was performed to assess the normality of data. The proportions were analyzed statistically using the Chi^2^ and Fisher’s exact tests. A multivariate regression analysis was used to evaluate independent risk variables for newborn death in-hospital after adjusting for confounding factors. A Kaplan–Meier probability curve was plotted for independent risk factors to estimate the probability of death for these categories. The criterion for significance was fixed at 0.05. 

## 3. Results

### 3.1. Preterm Births vs. Full-Term Births

Table 1 presents the comparison of maternal and neonatal characteristics between the group of preterm births and the group of full-term pregnancies. Among maternal characteristics, it was observed that gestational age, duration of hospital stay, and proportion of high obstetrical risk pregnancies were statistically significantly different between comparison groups. The gestational age in the preterm group was 29.5 weeks, compared with 37.8 weeks (*p*-value < 0.001). The duration of hospitalization was significantly longer in preterms, with a median of 5.0 days, compared with a median of 3.3 days in the full-term group (*p*-value = 0.001). There was 81.4% of women at high obstetrical risk in the group of preterm births, compared with only 17.5% among full-term births (*p*-value < 0.001). All maternal comorbidities included in the study were in higher proportions within the cases associated with prematurity, and they were all statistically significant (preeclampsia, thrombophilia, anemia, peripartum infection, and other maternal infections).

Among neonatal characteristics (Table 1), an abnormal APGAR score was identified in 68.3% of premature newborns, compared with 18.2% of full-term newborns (*p*-value < 0.001). Birth weight was significantly lower in the premature group, with an average of 1503 g, compared with 2597 g in the full-term group (*p*-value < 0.001). Most preterm deliveries were C-sections (67.4% vs. 34.8%, *p*-value < 0.001). As a consequence of prematurity, the number of NICU admissions and resuscitation requirements were statistically significantly higher. Preterm newborns had a median of 16.9 days of hospitalization, compared with 3.7 days for those born full-term (*p*-value < 0.001). Therefore, the mortality in the group of preterms was statistically significantly higher (9.5% vs. 1.2%, *p*-value < 0.001). Lastly, the neonates born before term required significantly more medication therapy with steroids and antibiotics, as well as a higher need for surfactant therapy (24.1% vs. 10.4%, *p*-value < 0.001).

### 3.2. Four-Year Analysis

The four-year comparison of preterm newborns between the pre-pandemic and pandemic periods is described in Table 2. Although there were not many significant differences between characteristics of premature births and full-term newborns, it was determined that during the first 24 months of the COVID-19 pandemic, mothers who gave birth before term required a longer hospitalization, from a median of 4.2 days before the pandemic, to 6.7 days during the pandemic (*p*-value = 0.003). Anemia was also significantly different between the four years that were analyzed, although without a variation associated with the pandemic, since the highest prevalence of maternal anemia was in 2018 (65.2%, *p*-value = 0.042). Among neonatal characteristics, we did not identify any significant differences, although neonatal therapy with surfactant, steroids, and antibiotics was statistically higher during the pandemic.

**Figure 2:**
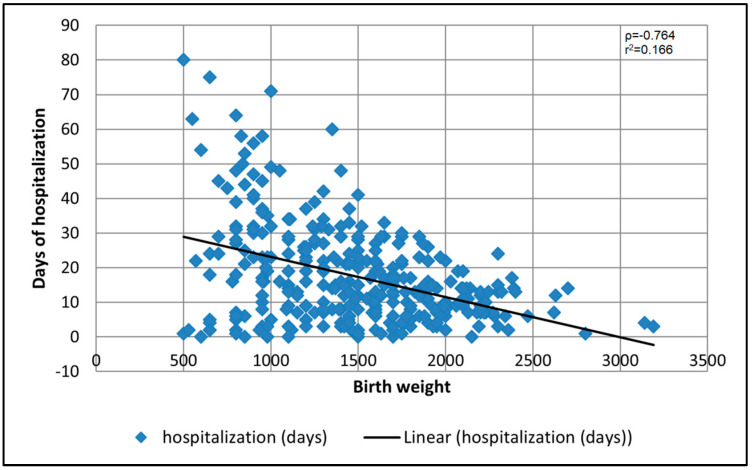
Dispersion chart with regression line of correlation between neonatal birth weight and hospitalization days.

The Pearson correlation coefficient between birth weight and the number of hospitalization days was strong and negative (ρ = −0.764, *p*-value < 0.001), while the goodness-of-fit measure for the linear regression model was also significant at the 95% significance threshold (r^2^ = 0.166, *p*-value = 0.030), as represented in Figure 2.

### 3.3. Risk Factor Analysis

The multivariate risk factor analysis presented in Table 3 determined that maternal age above 35 years old, twin births, low gestational age, preeclampsia, and peripartum infection were significant independent risk factors for NICU admission (age, OR = 1.59; twin births, OR = 1.14; low gestational age, OR = 1.66; preeclampsia, OR = 2.33; peripartum infection, OR = 2.25). The same risk factors, except for twin births, were significantly associated with in-hospital neonatal mortality (age, OR = 1.40; low gestational age, OR = 1.27; preeclampsia, OR = 1.24; peripartum infection, OR = 1.93).

**Figure 3:**
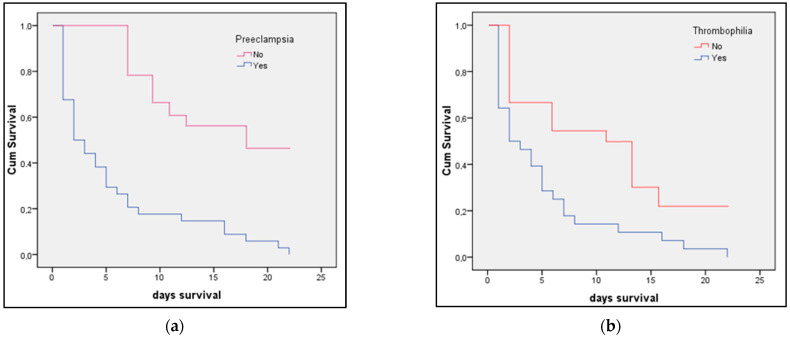
(**a**,**b**) Kaplan–Meier plots of preterm newborn survival (in days) from mothers with preeclampsia or thrombophilia compared with newborns from mothers without preeclampsia or thrombophilia.

**Figure 4:**
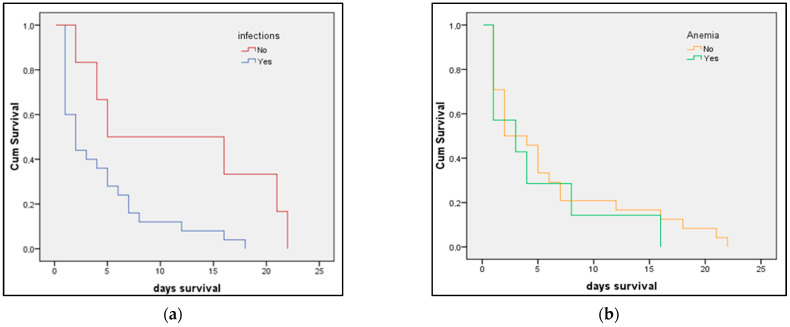
(**a**,**b**) Kaplan–Meier plots of preterm newborn survival (in days) from mothers with anemia or peripartum infections compared with newborns from mothers without anemia or peripartum infections.

**Figure 5:**
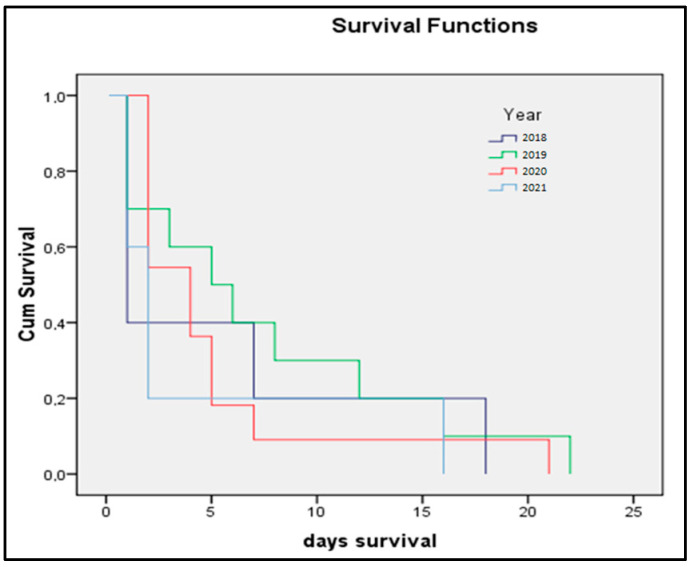
Kaplan–Meier plot of preterm newborn survival (in days)—4-year comparison.

The Kaplan–Meier survival prediction for premature infants presented in Figure 3a,b identified preeclampsia as determining significantly lower survivability for newborns (log-rank *p*-value = 0.009). The survival analysis presented in Figure 4a,b showed that newborns from mothers with peripartum infection also have significantly lower survival (log-rank *p*-value = 0.040). The survival analysis in Figure 5 represents the probability of survival among preterm newborns, and it was determined that there is no significant difference between the pre-pandemic and pandemic periods (log-rank *p*-value = 0.836). 

## 4. Discussion

The current study identified that advanced maternal age, twin pregnancy, low gestational age, preeclampsia, and peripartum infections were the predominant maternal risk factors for NICU admission. Additionally, these characteristics increased the chance of mortality, despite the neonates’ access to competent medical care and enhanced life support. Our results are also consistent with previous research indicating that congenital abnormalities were an important cause of NICU admission and mortality among premature newborns, although several maternal risk factors were identified as having lower but significant odds for the same complications [29,30]. Although numerous congenital defects are preventable, they continue to be significant causes of infant mortality. Gestational age at birth is another strong predictor of neonatal mortality. However, several congenital malformations may be prevented by prenatal folic acid and multivitamin supplementation, which has been shown to reduce the prevalence of abnormalities such as neural tube defects [31]. 

There is a considerable difference in mortality between infants delivered at 24 weeks and full-term, indicating the important influence of prematurity on neonatal survival. Increased preterm births result in an increase in newborn mortality. Congenital abnormalities, delivery trauma, birth asphyxia, and hospital-acquired infection are other reasons for newborn mortality that were previously reported but not evaluated in the current study [32]. A recent national investigation showed many important risk factors, including preterm, low birth weight, mother’s age under 20 years, a history of newborn mortality or stillbirth, preeclampsia, insufficient prenatal care, congenital malformations, and gestational age less than 37 weeks [33]. 

Assessing the number and etiology of these significant occurrences, as well as their risk factors, begins with classifying and reporting neonatal fatalities appropriately. A regionalized and integrated perinatal network plan should be established to minimize perinatal morbidity and death and to enhance preterm delivery and other high-risk newborn survival. Mortality data should be accessible by geographic region, rural or urban, site of death, date, underlying cause, and other variables such as socioeconomic status [34]. This may assist stakeholders in establishing priorities, planning, and monitoring progress. 

Similar to our results, earlier research has shown that immaturity, as measured by gestational weeks at delivery, is a major predictor of newborn mortality [35]. Neonatal death rates may vary dramatically between preterm newborns and their full-term counterparts born at 39–40 weeks gestation. The most prevalent risk factors for newborn mortality, identified by other studies, were prematurity, gestational age of fewer than 37 weeks, low birth weight, and multiple pregnancies [36,37]. Low birth weight may occur as a consequence of prenatal growth restriction or preterm delivery, both of which are related to placental malfunction and consequent adverse fetal outcomes [38]. Congruent with the current study’s findings, previous research has established that emergency C-section is associated with an increased risk of neonatal mortality and that cesarean section rates greater than 10% do not result in a reduction in mortality and should therefore be avoided whenever possible [39]. 

Several other background factors were not analyzed in the current study. It was previously observed that neonatal mortality rate did not differ significantly by mother’s age, income, or employment status; on the other hand, mothers with a high school education or less were associated with a higher rate of neonatal deaths, likely due to a lack of awareness about how and when to seek medical care, particularly in emergency situations [1,40]. Some evidence indicates that advanced maternal age is connected with placental malfunction, which may raise the risk of newborn deaths and stillbirths or exacerbate pre-existing maternal medical conditions. Additionally, newborns born to wealthy households with a high educational level have a better probability of survival than those born to more impoverished homes with a lower educational level [41].

## 5. Conclusions

Even though the majority of neonatal fatalities are preventable with effective treatments such as access to emergency obstetric and neonatal care, certain severe risk factors predispose newborns to severe complications and death, despite access to critical healthcare services. Nonetheless, stakeholders must understand the socioeconomic and geographic patterns of newborn mortality in order to expand access to effective therapies with a particular emphasis on high-risk populations. This will guarantee that every pregnant woman and newborn has the same level of access to life-saving treatments.

## Figures and Tables

**Table 1:** Comparison of maternal and neonatal characteristics between the group of preterm births and the group of full-term pregnancies.

	Preterm (*n* = 328)	Full-Term (*n* = 422)	*p*-Value *
**Maternal background**			
*Age (≥35 years)*	81 (24.7%)	115 (27.3%)	0.429
*Weight at birth (>25 kg/m^2^)*	74 (22.6%)	79 (18.7%)	0.195
*Twin birth*	40 (12.2%)	49 (11.6%)	0.806
*Gestational age*	29.5 ± 2.4	37.8 ± 4.9	<0.001
Days of hospitalization **	5.0 (2.9–7.1)	3.3 (1.9–6.0)	0.001
*High obstetrical risk (n–%)*	267 (81.4%)	74 (17.5%)	<0.001
**Maternal comorbidities**			
*Preeclampsia*	12 (3.7%)	6 (1.4%)	0.047
*Thrombophilia*	20 (6.1%)	13 (3.1)	0.045
*Anemia*	184 (56.1%)	123 (29.1%)	<0.001
*Peripartum infection*	82 (25.0%)	24 (5.7%)	<0.001
*Other maternal infections*	68 (20.7%)	28 (6.6%)	<0.001
**Neonatal characteristics**			
*Gender (male)*	172 (52.4%)	198 (46.9%)	0.133
*Abnormal APGAR score*	224 (68.3%)	77 (18.2%)	<0.001
Birth weight *** (grams)	1503 ± 494	2597 ± 606	<0.001
*In vitro fertilization*	28 (8.5%)	33 (7.8%)	0.721
*Delivery type (C-section)*	221 (67.4%)	147 (34.8%)	<0.001
*Infection after membrane rupture*	71 (21.6%)	76 (18.0%)	0.213
*Congenital abnormalities*	14 (4.3%)	8 (1.9%)	0.056
*Severe prematurity*	39 (11.9%)	-	-
*NICU admission*	42 (12.8%)	11 (2.6%)	<0.001
*Resuscitation*	38 (11.6%)	19 (4.5%)	<0.001
Days of hospitalization **	16.9 (14.3–19.8)	3.7 (1.4–5.9)	<0.001
Days of NICU stay **	8.3 (5.1–12.8)	7.7 (4.9–11.7)	0.084
*Mortality*	31 (9.5%)	5 (1.2%)	<0.001
**Neonatal therapy**			
*Surfactant*	79 (24.1%)	44 (10.4%)	<0.001
*Steroids*	52 (15.9%)	38 (9.0%)	0.004
*Antibiotics*	260 (79.3%)	194 (46.0%)	<0.001

* Chi-square or Fisher’s exact test; ** Data represented as median (IQR); *** In correlation with gestational age; APGAR—Appearance, Pulse, Grimace, Activity, Respiration; NICU—Neonatal Intensive Care Unit.

**Table 2:** Four-year comparison of preterm birth characteristics.

	2018 (*n* = 48)	2019 (*n* = 115)	2020 (*n* = 86)	2021 (*n* = 79)	*p*-Value *
**Maternal background**					
*Age ≥ 35 years*	8 (16.7%)	29 (25.2%)	20 (23.3%)	24 (30.4%)	0.369
*Weight at birth (>kg/m^2^)*	10 (20.8%)	22 (19.1%)	20 (23.6%)	18 (22.7%)	0.447
*Twin birth (>25 kg/m^2^)*	6 (12.5%)	14 (12.2%)	16 (18.6%)	4 (5.1%)	0.070
*Gestational age*	29.9 ± 2.3	29.4 ± 2.3	29.6 ± 2.4	29.7 ± 2.3	0.105
Days of hospitalization **	4.3 (1.9–6.3)	4.1 (1.9–6.0)	6.6 (2.8–7.2)	6.8 (2.9–7.4)	0.003
*High obstetrical risk (n–%)*	40 (83.3%)	89 (77.4%)	81 (94.2%)	57 (72.2%)	0.672
**Maternal comorbidities**					
*Preeclampsia*	2 (4.2%)	4 (3.5%)	3 (3.5%)	3 (3.8%)	0.996
*Thrombophilia*	3 (6.3%)	11 (9.6%)	3 (3.5%)	3 (3.8%)	0.244
*Anemia*	21 (43.8%)	75 (65.2%)	43 (50.0%)	45 (57.0%)	0.042
*Peripartum infection*	14 (29.2%)	24 (20.9%)	25 (29.1%)	19 (24.1%)	0.515
*Other maternal infections*	14 (29.2%)	25 (21.7%)	18 (20.9%)	11 (13.9%)	0.223
**Neonatal characteristics**					
*Gender (male)*	19 (39.6%)	65 (56.5%)	46 (53.5%)	42 (53.2%)	0.261
*Abnormal APGAR score*					
Birth weight ***(grams)	1521 ± 517	1416 ± 449	1473 ± 537	1480 ± 496	0.246
*In vitro fertilization*	3 (6.3%)	10 (8.7%)	12 (14.0%)	3 (3.8%)	0.120
*Delivery type (C-section)*	34 (70.8%)	79 (68.7%)	50 (58.1%)	58 (73.4%)	0.172
*Infection after membrane rupture*	10 (20.8%)	24 (20.9%)	19 (22.1%)	19 (24.0%)	0.956
*Congenital abnormalities*	2 (4.2%)	6 (5.2%)	3 (3.5%)	3 (3.8%)	0.934
*Severe prematurity*	6 (12.5%)	10 (8.7%)	13 (15.1%)	10 (12.6%)	0.567
*NICU admission*	3 (6.2%)	13 (11.3%)	13 (15.1%)	9 (11.4%)	0.495
*Resuscitation*	0 (0.0%)	15 (13.0%)	14 (16.3%)	9 (11.4%)	0.038
Days of hospitalization **	15.9 (11.5–20.3)	20.3 (17.4–23.3)	16.7 (14.0–19.5)	17.5 (15.9–19.0)	0.053
Days of NICU stay **	8.1 (5.3–12.1)	9.0 (5.8–13.4)	8.6 (5.5–12.3)	8.5 (5.2–12.4)	0.496
*Mortality*	5 (10.4%)	10 (8.7%)	11 (12.8%)	5 (6.3%)	0.542
**Neonatal therapy**					
*Surfactant*	5 (10.4%)	25 (21.7%)	24 (27.9%)	25 (31.6%)	0.038
*Steroids*	0 (0.0%)	3 (2.6%)	20 (23.3%)	29 (36.7%)	<0.001
*Antibiotics*	24 (50.0%)	79 (68.7%)	81 (94.2%)	76 (96.2%)	<0.001

* Chi-square or Fisher’s exact test; ** Data represented as median (IQR); *** In correlation with gestational age; APGAR—Appearance, Pulse, Grimace, Activity, Respiration; NICU—Neonatal Intensive Care Unit.

**Table 3:** Maternal risk factor analysis for in-hospital neonatal intensive care unit admission and mortality in premature newborns.

Risk Factors	NICU Admission (OR–95% CI)	*p*-Value	Mortality (OR–95% CI)	*p*-Value
*Age ≥ 35 years*	1.59 (1.21–2.37)	0.012	1.40 (1.06–1.99)	0.041
*Weight at birth*	1.02 (0.84–1.23)	0.294	0.93 (0.82–1.06)	0.192
*Twin births*	1.14 (1.02–1.36)	0.047	1.02 (0.82–1.25)	0.298
*Low gestational age*	1.66 (1.23–2.11)	0.001	1.27 (1.02–1.88)	0.017
*High obstetrical risk (n–%)*	1.19 (0.91–1.32)	0.402	1.01 (0.88–1.33)	0.194
*Preeclampsia*	2.33 (1.86–3.18)	0.001	1.24 (1.09–1.76)	0.037
*Thrombophilia*	1.15 (0.97–1.43)	0.316	0.98 (0.75–1.04)	0.339
*Peripartum infection*	2.25 (1.46–3.23)	0.001	1.93 (1.16–2.83)	0.009
*Anemia*	1.12 (0.93–1.32)	0.422	1.05 (0.83–1.41)	0.510
*Other maternal infections*	1.07 (0.92–1.40)	0.294	1.01 (0.84–1.29)	0.318

NICU—Neonatal Intensive Care Unit; OR—Odds Ratio; CI—Confidence Interval.

## Data Availability

The data presented in this study are available on request from the corresponding author.
